# Th2/1 Hybrid Cells Occurring in Murine and Human Strongyloidiasis Share Effector Functions of Th1 Cells

**DOI:** 10.3389/fcimb.2017.00261

**Published:** 2017-06-20

**Authors:** Cristin N. Bock, Subash Babu, Minka Breloer, Anuradha Rajamanickam, Yukhti Boothra, Marie-Luise Brunn, Anja A. Kühl, Roswitha Merle, Max Löhning, Susanne Hartmann, Sebastian Rausch

**Affiliations:** ^1^Department of Veterinary Medicine, Institute of Immunology, Freie Universität BerlinBerlin, Germany; ^2^National Institutes of Health-NIRT-International Center for Excellence in ResearchChennai, India; ^3^Laboratory of Parasitic Diseases, National Institutes of Allergy and Infectious Diseases, National Institutes of HealthBethesda, MD, United States; ^4^Section for Molecular Biology and Immunology, Bernhard Nocht Institute for Tropical MedicineHamburg, Germany; ^5^Medical Department, Division of Gastroenterology, Infectiology and Rheumatology/Research Center ImmunoSciences, Charité-University Medicine BerlinBerlin, Germany; ^6^Department of Veterinary Medicine, Institute for Veterinary Epidemiology and Biostatistics, Freie Universität BerlinBerlin, Germany; ^7^Experimental Immunology, Department of Rheumatology and Clinical Immunology, Charité-University Medicine BerlinBerlin, Germany; ^8^Pitzer Laboratory of Osteoarthritis Research, German Rheumatism Research Center (DRFZ), Leibniz InstituteBerlin, Germany

**Keywords:** nematode, strongyloides, Th2, hybrid, T-bet, GATA-3, co-expression, cytokines

## Abstract

Infections by the soil-transmitted threadworm *Strongyloides stercoralis* affect 30–100 million people worldwide, predominantly in tropic and sub-tropic regions. Here we assessed the T helper cell phenotypes in threadworm-infected patients and experimental murine infections with focus on CD4^+^ T cells co-expressing markers of Th2 and Th1 differentiation. We show that mice infected with the close relative *S. ratti* generate strong Th2 responses characterized by the expansion of CD4^+^ GATA-3^+^ cells expressing IL-4/-5/-13 in blood, spleen, gut-draining lymph nodes, lung and gut tissue. In addition to conventional Th2 cells, significantly increased frequencies of GATA-3^+^T-bet^+^ Th2/1-hybrid cells were detected in all organs and co-expressed Th2- and Th1-cytokines at intermediate levels. Assessing the phenotype of blood-derived CD4^+^ T cells from South Indian patients infected with *S. stercoralis* and local uninfected control donors we found that GATA-3 expressing Th2 cells were significantly increased in the patient cohort, coinciding with elevated eosinophil and IgE/IgG4 levels. A fraction of IL-4^+^CD4^+^ T cells simultaneously expressed IFN-γ hence displaying a Th2/1 hybrid phenotype. In accordance with murine Th2/1 cells, human Th2/1 cells expressed intermediate levels of Th2 cytokines. Contrasting their murine counterparts, human Th2/1 hybrids were marked by high levels of IFN-γ and rather low GATA-3 expression. Assessing the effector function of murine Th2/1 cells *in vitro* we found that Th2/1 cells were qualified for driving the classical activation of macrophages. Furthermore, Th2/1 cells shared innate, cytokine-driven effector functions with Th1 cells. Hence, the key findings of our study are that T helper cells with combined characteristics of Th2 and Th1 cells are integral to immune responses of helminth-infected mice, but also occur in helminth-infected humans and we suggest that Th2/1 cells are poised for the instruction of balanced immune responses during nematode infections.

## Introduction

Infections by helminths affect approximately 2 billion people globally, with soil-transmitted worms being the most prevalent. Infections with the threadworm *S. stercoralis* are currently estimated to afflict approximately 30–100 million people worldwide and are mostly asymptomatic (Puthiyakunnon et al., 2014). However, when unrecognized, the infection bears the risk of developing into a life-threatening condition in states of immune suppression (Weatherhead and Mejia, 2014).

Infections with parasitic nematodes lead to the instruction of type 2 immune responses marked by the differentiation of naïve CD4^+^ T cells into T helper type 2 (Th2) cells (Anthony et al., 2007). These are characterized by the expression of the lineage-specifying transcription factor GATA-3 resulting in the competence to produce the effector cytokines interleukin (IL)-4, IL-5 and IL-13 (Zheng and Flavell, 1997; Zhu et al., 2010). Animal studies show that Th2 responses are central to the control of enteric helminth infections by orchestrating a broad spectrum of defense mechanisms, such as the production of Th2-driven antibody subclasses, specialized macrophage effector programs and physiological changes like intestinal goblet cell hyperplasia, mucus hyper-secretion and intensified intestinal smooth muscle contractions (Finkelman et al., 2004; Patel et al., 2009; Harris and Gause, 2011; Allen and Sutherland, 2014). While primary infections are often long lasting, the resulting Th2-dominated immunological environment is highly effective in restricting experimental re-infection under laboratory conditions (Dawkins and Grove, 1981; Urban et al., 1991; Finkelman et al., 1997; Anthony et al., 2007; Eschbach et al., 2010). Many species, however, manage to re-infect their host, as exemplified by hookworms (*Necator, Ancylostoma*), whipworms (*Trichuris*), and *Ascaris* repeatedly infecting humans by tissue migrating larvae or the ingestion of infective eggs, respectively (Turner et al., 2003, 2008; Quinnell et al., 2004; Figueiredo et al., 2010). *S. stercoralis* is unique as the parthenogenic larvae are able to develop further into adults in the infected host, leading to multiple and potentially lifelong circles of autoinfection (Weatherhead and Mejia, 2014).

We have previously shown the induction of a stably differentiated hybrid T helper population with combined characteristics of Th2 and Th1 cells at the single cell level, namely the co-expression of GATA-3 and Th2 cytokines together with the lineage-specifying transcription factor and signature cytokine of Th1 cells, T-bet and IFN-γ, in experimental helminth infections. These cells, while being able to support both Th2 and Th1 immune responses, display a quantitatively reduced potential for Th2- as well as Th1-associated effector functions (Peine et al., 2013). We asked whether such Th2/1 cells also occur in helminth-infected patients and hence investigated T helper cell responses in patients infected by *S. stercoralis* in South India. Experimental infections with the murine model *S. ratti* were employed to assess whether the development and proportions of Th2/1 hybrid cells differ depending on parasite burden and phase of infection and to collect more detailed information about the prevalence of Th2/1 hybrid, conventional Th2 and Th1 cells in different organs affected by the parasite during its life cycle. Furthermore, we aimed to assess if Th2/1, similar to Th1 cells present in higher numbers, may serve as a source for IFN-γ sufficient for the instruction of classical macrophage activation.

We show that Th2/1 hybrid cells co-expressing IL-4 and IFN-γ are not restricted to a considerable range of murine helminth infections, but are also detectable in *S. stercoralis* infected patients. In mice, the proportion of Th2/1 hybrids within Th2 cells was independent of worm burden or phase of infection, but Th2/1 cells were most prominent in spleen and small intestine. To our knowledge, we show for the first time that human Th2/1 hybrid cells are marked by high IFN-γ and low GATA-3 expression, contrasting the IFN-γ/GATA-3 intermediate phenotype of their murine counterparts. Functionally, murine Th2/1 hybrid cells shared effector aspects with Th1 cells in producing IFN-γ in response to cytokine triggers and the ability to drive classical macrophage activation.

## Materials and methods

### Ethics statement and study population

All individuals were examined as part of a natural history study protocol approved by Institutional Review Boards of the National Institute of Allergy and Infectious Diseases, USA and the National Institute for Research in Tuberculosis, India (ClinicalTrials.gov identifiers NCT00375583, and NCT00001230), and informed written consent was obtained from all participants.

We studied a total of 74 individuals comprising 34 clinically asymptomatic, *S. stercoralis*-infected individuals (Inf) and 40 uninfected, healthy individuals with endemic normal status (EN) in Tamil Nadu, South India (Tables 1, 2). All individuals were recruited from a rural population by screening of individuals for helminth infection by stool microscopy and serology. Inclusion criteria were age of 18 to 65 years and willingness to give blood and stool samples for examination; exclusion criteria were past anthelmintic treatment, other helminth infections, or HIV infection. Strongyloides infection was diagnosed by the presence of IgG antibodies to the recombinant *Strongyloides* antigen, NIE, as described previously (Bisoffi et al., 2014; Buonfrate et al., 2015). None of the individuals had lymphatic filariasis verified with TropBio Og4C3 enzyme-linked immunosorbent assay (ELISA) (Trop Bio Pty. Ltd., Townsville, Queensland, Australia) or other intestinal helminths (based on stool microscopy). None of the tested individuals suffered from acute tuberculosis, analyzed via QuantiFERON TB Gold-in-Tube enzyme-linked immunosorbent assay (ELISA) (Cellestis).

**Table 1:** Demographic profile of infected and uninfected cohorts.

**Parameter**	**Value for the group**		
	**Infected (*n* = 34)**	**Uninfected (*n* = 40)**
No. of male subjects	16	21
No. of female subjects	18	19
Mean age (range [year])	34 (19–58)	33 (18–60)
Clinical status	Healthy	Healthy
Symptom(s)	None	None
Socioeconomic status	Rural workers	Rural workers
NIE ELISA result	Positive	Negative
Results of stool examination for *S. stercoralis*	Positive	Negative
Presence of other helminth infections	Negative	Negative

**Table 2:** Hematological profile of infected and uninfected cohorts.

**Factor^a^**	**GM (range) for the group**		
	**Infected (*n* = 34)**	**Uninfected (*n* = 40)**	***P*-value^b^**
Hb (g/dL)	13.07 (7.6–16.9)	51.77 (8.7–174)	NS
RBC (10^6^ /μl)	4.68 (3.7–6)	4.80 (3.5–6.46)	NS
WBC (10^3^ /μl)	8.51 (5.1–13.8)	8.55 (5.3–13.3)	NS
HCT (%)	39.47 (25–51)	40.35 (27–54)	NS
PLT (10^3^ /μl)	267.5 (159–413)	287.1 (137–446)	NS

a *Hb, hemoglobin; RBC, red blood cells; WBC, white blood cells; HCT, hematocrit; PLT, platelets*.

b *NS, not significant*.

All infected individuals were treated with single doses of ivermectin and albendazole. All uninfected individuals were anti-*Strongyloides* NIE negative and negative for filarial and other intestinal helminths as well as acute tuberculosis.

### Hematological parameters

Hemograms were performed on all individuals using the Act-5 Diff hematology analyzer (Beckman Coulter, Brea, CA, USA).

### PBMC isolation and *in vitro* culture

Heparinized blood was centrifuged at 400 × g for 10 min, plasma separated and stored at 4°C. Samples were refilled with RPMI-1640 and PBMC isolated by Ficoll diatrizoate gradient centrifugation (LSM; ICN Biomedicals). Cells were then washed, resuspended in freezing medium (RPMI 1640, 10% DMSO, 45% heat inactivated FCS, Harlan Bioproducts) and stored in liquid nitrogen until usage. For *in vitro* culture the cryopreserved cells were thawed gently, washed twice and cultured with RPMI 1640 medium supplemented with penicillin-streptomycin (100 U and 100 μg/ml, respectively), L-glutamine (2 mM), 1% NEAA MEM (all from PAN–Biotech, Aidenbach, Germany) and 5% heat inactivated AB human serum (Biochrom, Berlin, Germany). For stimulations and intracellular staining cells were counted using trypan blue and adjusted to 1 × 10^7^ cells/ml. 2 × 10^6^ cells/well were placed on round-bottom 96 well tissue culture plates, stimulated with phorbol 12-myristate 13-acetate (PMA) and ionomycin (both Sigma-Aldrich, MO, USA) at concentrations of 25 ng/ml and 0.5 μg/ml or media alone and incubated for 4 h at 37°C. Brefeldin A solution (10 μg/ml, eBioscience, CA, USA) was added after 30 min. After 4 h cells were washed, stained and analyzed as given below.

### Elisa

Plasma levels of total IgE, IgG1, IgG3, and IgG4 were evaluated using Ready-Set-Go! ELISA kits (eBioscience) according to the manufacturer's instructions. All samples were run in duplicates.

### Animal experimentation, mice and parasites

Animal experiments were performed in accordance with the National Animal Protection Guidelines and approved by Federal Health Authorities of the States of Hamburg (permission number 55/13). The *S. ratti* life cycle was maintained in Wistar rats purchased from Charles River (Sulzfeld, Germany) as described earlier (Eschbach et al., 2010). Female C57BL/6 mice were bred in house at the Bernhard Nocht Institute Hamburg, Germany. Mice were kept in individually ventilated cages under specific pathogen-free (SPF) condition, infected by 200 or 2,000 *S. ratti* iL3 in the hind footpad and sacrificed by isoflurane inhalation followed by cervical dislocation at day 10 or 20 post infection at 8–10 weeks of age. Parasite burdens were examined at day 6 post infection in faces via quantification of *S. ratti* 28S ribosomal RNA using real-time quantitative PCR as described elsewhere (Nouir et al., 2012).

### Preparation of single cell suspension

Spleens and mesenteric lymph nodes (mLN) were isolated and placed in cold RPMI 1640 wash medium containing 1% FCS, 100 U/ml penicillin and 100 μg/ml streptomycin (all from PAN – Biotech, Aidenbach, Germany) and then forced through 70 μm cell strainers (BD Bioscience, San Jose, CA) to obtain single cell suspensions. Erythrocytes in spleen cell suspension were lysed using ACK buffer containing 150 mM NH_4_Cl, 0.1 mM KHCO_3_ and 0.1 mM Na_2_EDTA, pH 7.2 for 5 min on ice followed by two washing steps. Small intestinal lamina propria (siLP) cells were isolated as described earlier (Strandmark et al., 2016).

Lungs were perfused and rinsed with 0.9% NaCl until the tissue turned white before isolation of single cells. The lung was rinsed in ice-cold RPMI 1640 containing 10% FCS. Tissue was then transferred onto petri dishes, cut in small pieces (~ 2 mm) and subsequently added to 10 ml PBS containing 0.1265 U/ml collagenase D and DNase (0.15 mg/ml, both Sigma-Aldrich, Steinheim, Germany) followed by incubation in a tube shaker water bath (250 rpm, 37°C) for 1 h. Digested lung tissues were forced through 40 μm cell strainers, washed twice and erythrocytes lysed using ACK buffer followed by two washing steps.

For the isolation of PBMC, whole blood was diluted 2:1 with RPMI 1640 containing 10% FCS and layered onto Pancoll (density of 1.077 g/ml, PAN-Biotech, Aidenbach, Germany), centrifuged and washed twice with PBS.

Cells of all tissues were resuspended in RPMI 1640 containing 10% FCS, counted using a CASY automated cell counter (Roche-Innovatis, Reutlingen, Germany) and were adjusted to 1 × 10^7^ cells/ml. Cells were plated on round-bottom 96 well tissue culture plates (Costar, Corning Inc., NY, USA), stimulated with PMA and ionomycin at concentrations of 50 ng/ml and 1 μg/ml and incubated for 30 min at 37°C, followed by addition of Brefeldin A (10 μg/ml) and further stimulation for 2.5 h.

### Flow cytometry

Human cells were stained with fixable viability dyes (eFluor780 or eFluor506 eBioscience), fixed and permeabilized using the eBioscience Foxp3 staining kit and stained intracellularly for the following markers: CD3 (BV510, clone OKT3), CD4 (PerCP, clone SK3), CD45RO (FITC, clone UCHL1), IL-4 (PE, MP4-25D2), IL-5 (PE, clones MP4-25D2, TRFK5), IL-13 (FITC, clone PVM13-1), IFN-γ, (eF450, clone 4S.B3), GATA-3 (eF660, clone TWAJ) and T-bet (PE-Cy7, clone eBio4B10). Murine cells were stained with fixable viability dye (eFluor780, eBioscience) and subsequently fixed and permeabilized using the Foxp3 staining kit and stained intracellularly for the following markers: CD4 (PerCP, clone RM4-5), GATA-3 (eFluor660, clone TWAJ), T-bet (PE-Cy7, clone eBio4B10), IL-4 (PE, clone 11B11), IL-13 (Alexa488, clone eBio13A), IL-5 (PE, clone TRFK5), IFN-γ (eFluor450, clone XMG1.2). All antibodies were from eBioscience, BioLegend and BD Biosciences.

### *In vitro* T helper subset differentiation

Cells from spleens and peripheral lymph nodes of naïve C57BL/6 mice were stained for CD4 (BrilliantViolet 510, clone RM4-5), CD62-L (APC-eFluor780, clone MEL-14), CD44 (PE, clone IM7) and CD25 (APC, clone 61.5) (all antibodies from eBioscience and BioLegend) and naïve CD4^+^CD25^−^CD62-L^high^CD44^neg^ cells were isolated on a FACS Aria cell sorter (BD Bioscience). Cells were plated on 48 well plates (Costar) coated with anti-CD3/anti-CD28 antibodies (1 μg/ml each, clones 145-2C11 and 37.51, from BD Biosciences). Th1 differentiation was induced by addition of recombinant murine IFN-γ and IL-12 (10 ng/ml each, PeproTech) and 10 μg/ml anti-IL-4 (clone 11B11, BioLegend, San Diego, CA, USA). Th2 development was induced by addition of 30 ng/ml recombinant murine IL-4 (PeproTech) and anti-IFN-γ/anti-IL12/23 p40 antibodies (clones AN18, C17.8, BioLegend, 10 μg/ml each). Th2/1 hybrids were generated by addition of IFN-γ, IL-12 and IL-4 in the concentrations given above. Recombinant human IL-2 (10 ng/ml, PeproTech) was added to all cultures and replaced in fresh medium on day 3. Cells were analyzed for expression of T-bet, GATA-3, IFN-γ IL-4 and IL-13 after 5 to 6 days by intracellular antibody staining.

### T helper cell innate function

Cells isolated from mice infected with *S. ratti* for 10 days were stimulated with the following recombinant murine cytokines (all from PeproTech, Hamburg) or monoclonal antibodies (from BD Biosciences) for 6 h: IL-33 (100 ng/ml) and IL-7 (10 ng/ml), IL-18 and IL-12 (both 10 ng/ml), anti-CD3 (clone 145-2C11) and anti CD28 antibodies (clone 37.51, both 2 μg/ml). Controls were left untreated. Brefeldin A (10 μg/ml) was added after 30 min. Cells were then stained for CD4, GATA-3, T-bet, IL-13 and IFN-γ.

### Macrophage/T cell co-cultures and nitric oxide assay

Peritoneal cells were isolated from naïve C57BL/6 mice by flushing the peritoneal cavity with ice-cold PBS (20 mM EDTA, 0.2% BSA) followed by washing in RPMI. 3 × 10^5^ cells were plated on 96 well round-bottom plates (Costar) in 200 μl in RPMI and incubated at 37°C for 1 h. Non-adhering cells were removed by 3 washing steps with warm RPMI. 1 × 10^5^ *in vitro* generated Th1, Th2 or Th2/1 cells were added and co-cultured in 200 μl for 24 h. Lipopolysaccheride (LPS, L5529, Sigma) was added for co-stimulation at 0.5 μg/ml to some wells. Control cultures received 1 or 10 ng/ml recombinant murine IFN-γ. Nitrite concentrations in culture media were quantified by the Griess reaction.

### Histology

Proximal small intestinal tissue samples were fixed in formalin and histochemically stained with hematoxylin and eosin (H&E) to assess histomorphology and with periodic acid Schiff (PAS) for goblet cell quantification.

Enteritis was scored as described earlier (Rausch et al., 2009). Images were acquired using the AxioImager Z1 microscope (Carl Zeiss MicroImaging, Inc., Jena, Germany) at 100× magnification. All evaluations were performed in a blinded manner.

### Statistical analyses

All data were assessed for Gaussian distribution. Statistically significant differences between two groups were analyzed by using Student's *t*-test (parametric) or Mann-Whitney *U*-test (non-parametric) using GraphPad Prism 7 software (San Diego, CA). For multiple comparisons, the Kruskal-Wallis test with Dunn's correction was performed. *P*-values ≤ 0.05 were considered to indicate statistical significance.

The regression analyses were carried out using SAS 9.4. Quantitative variables were tested for normality (PROC UNIVARIATE) and when necessary transformed to logarithmic values. After graphic assessment of the assumption of linear relationships (PROC GPLOT) and assessment of correlations between variables (PROC CORR) associations between variables were investigated by multivariable linear models with the procedure PROC REG. Models with different independent factors were compared by using the adjusted *R*^2^ and the global F-statistics. The variable “group” as well as the main variable of the respecting analyses were always kept in the model, but other factors were removed in a backward selection according to the *p*-value and the change of *R*^2^. Model diagnostics included residues assessment (normality), test for multicollinearity and variance inflation. Observations with great influence (according to leverage hat-values or Cook's D) were excluded, if the model changed significantly after removing the observation. *P*-values ≤ 0.05 were considered to indicate statistical significance.

## Results

### *S. ratti* infection results in Th2 induction

**Figure 1:**
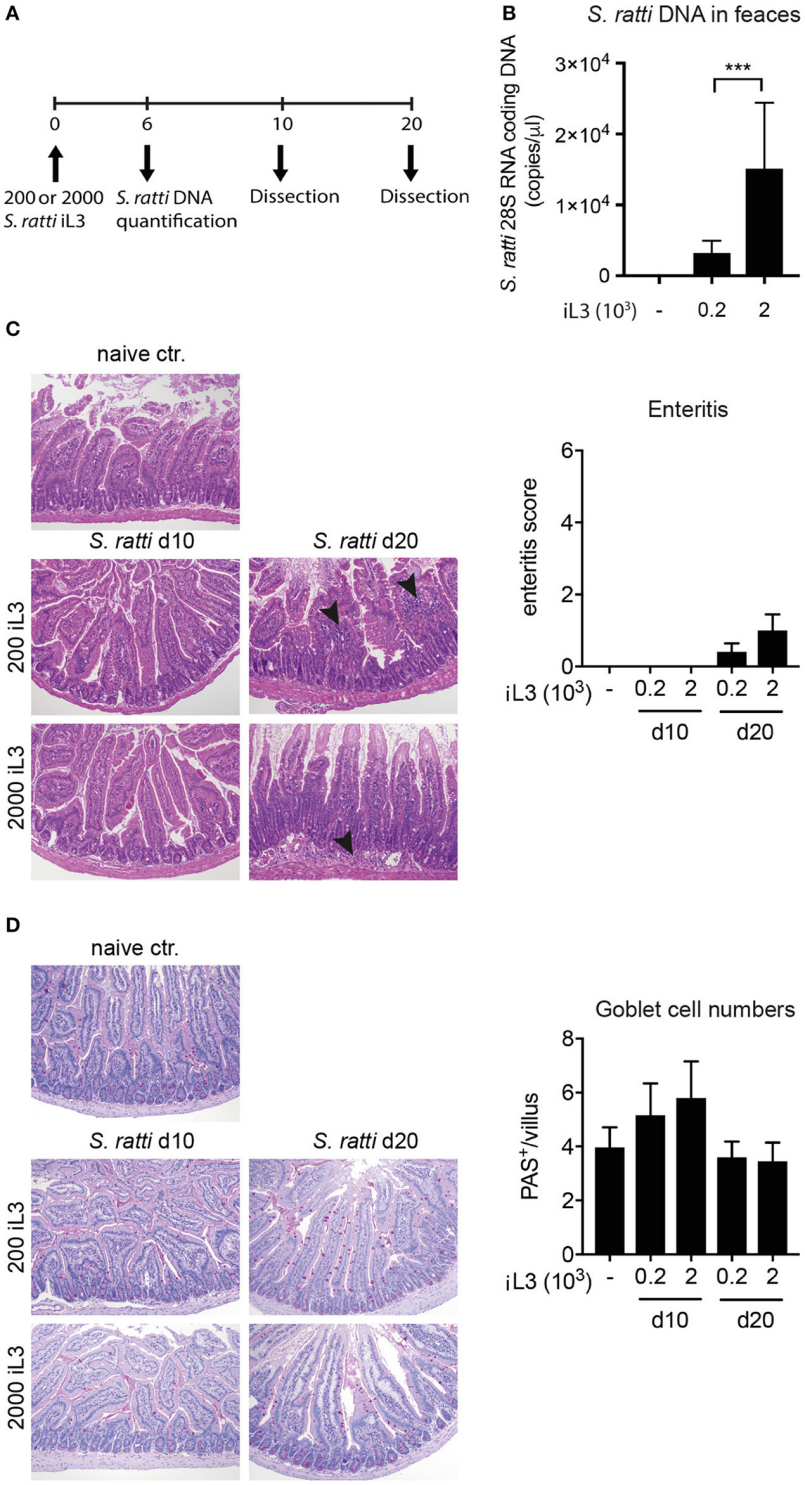
*S. ratti* infection leads to mild immunopathology in mice. **(A)** Experimental time line. C57BL/6 mice were infected with 200 or 2,000 *S. ratti* stage 3 larvae and dissected 10 or 20 days post infection. **(B)** Quantification of *Strongyloides* DNA at day 6 post infection. **(C)** Exemplary pictures of H&E stained proximal small intestinal cross sections. Magnification × 100. Mild cellular infiltrates in the lamina propria at day 20 are marked by arrow heads. Bar graph depicts enteritis scores of experimental groups. **(D)** Exemplary pictures of PAS stained proximal small intestinal cross sections. Magnification × 100. Mucus filled goblet cells appear magenta. Bar graph depicts quantification of goblet cells per villus in small intestinal cross sections. Mean + SD of *n* = 6 (naïve ctr.) and 5 (infected) mice is shown. Data originate from one out of two experiments with similar results. ^***^p < 0.005.

In order to assess the immune response to *S. ratti*, mice were infected with 200 or 2,000 infective stage 3 larvae and dissected at day 10 or 20 post infection (Figure 1A). Worm burdens were assessed at day 6 by quantification of *Strongyloides* DNA in faces. Expectedly and as shown before, mice infected with 2,000 larvae displayed significantly higher worm burdens than mice inoculated with 200 larvae (Figure 1B; Eschbach et al., 2010). Histological examination of proximal small intestinal tissue samples showed that the infection did not result in immunopathological changes by day 10, while some mice displayed mild signs of cellular infiltration at day 20 post infection (Figure 1C). Numbers of mucus-producing goblet cells were at best mildly and transiently, but not significantly increased by day 10 post infection (Figure 1D).

**Figure 2:**
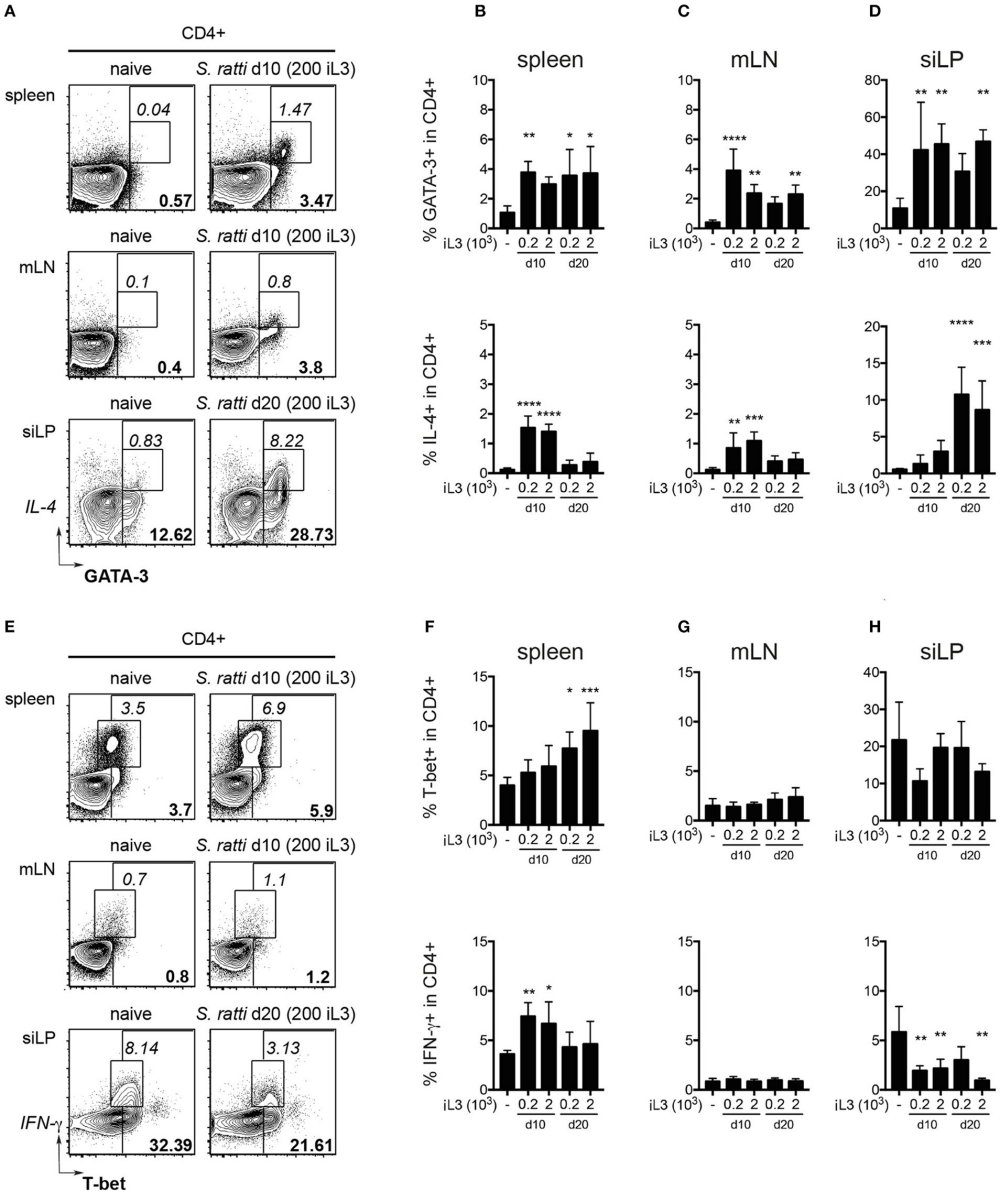
*S. ratti* infection leads to systemic and local Th2 response. Phenotypes of CD4^+^ T cells were assessed in mice infected with 200 or 2,000 *S. ratti* stage 3 larvae. **(A)** Exemplary flow cytometry plots of live CD4^+^ T cells derived from spleen, mesenteric lymph nodes (mLN) and small intestinal lamina propria (siLP) of an uninfected control and *S. ratti* infected mouse (200 iL3) at the depicted days post infection. Bold numbers report frequencies of GATA-3 expressing cells, italic numbers report frequencies of GATA-3^+^IL-4^+^ cells. **(B–D)** Frequencies of GATA-3^+^ cells (top) and IL-4^+^ cells (bottom) within live CD4^+^ T cells isolated from spleen **(B)**, mLN **(C)** and siLP **(D)** as detected after 4h of PMA/ionomycin stimulation. **(E)** Exemplary flow cytometry plots of live CD4^+^ T cells as described in **(A)**. Bold numbers report frequencies of T-bet expressing cells, italic numbers report frequencies of IFN-γ^+^ cells. **(F–H)** Frequencies of T-bet^+^ cells (top) and IFN-γ^+^ cells (bottom) within live CD4^+^ T cells isolated from spleen **(F)**, mLN **(G)** and siLP **(H)** detected after 4 h of PMA/ionomycin stimulation. Mean + SD of *n* = 5–6 (naïve ctr.) and 4–5 (infected) mice. Data from one out of two experiments with similar results are shown. ^*^p < 0.05, ^**^p < 0.01, ^***^p < 0.005, ^****^p < 0.001 comparing infected to naïve controls.

Expectedly, infected mice displayed significantly increased frequencies of CD4^+^ T cells expressing GATA-3 and IL-4 in spleen, gut-draining mesenteric lymph nodes (mLN) and small intestinal lamina propria (siLP) when compared to uninfected controls (Figures 2A–D). Th2 cells were also elevated in blood and lung tissue of infected mice (SI Figures 1A,B). T-bet^+^ Th1 cells tended to increase in the spleen of infected mice, reaching significance at day 20 post infection, while IFN-γ expression by CD4^+^ T cells from spleens was transiently elevated at day 10 (Figures 2E,F). Frequencies of T-bet expressing cells in mLN, siLP and other organs were similar in all groups (Figures 2G,H and SI Figures 1C,D) while IFN-γ responses in the small intestine were significantly reduced in infected mice (Figure 2H).

Collectively, *S. ratti* infection led to local and systemic Th2 responses confirming previous studies (Eschbach et al., 2010; Blankenhaus et al., 2011), while transiently elevated IFN-γ production and expansion of T-bet expressing Th1 cells was restricted to the spleen. Pathological changes and Th2-driven increases of goblet cells in the small intestine were only mild, pointing out the relatively asymptomatic nature of *S. ratti* infection in mice.

### Th2/1 hybrid cells occur in mice infected with *S. ratti*

**Figure 3:**
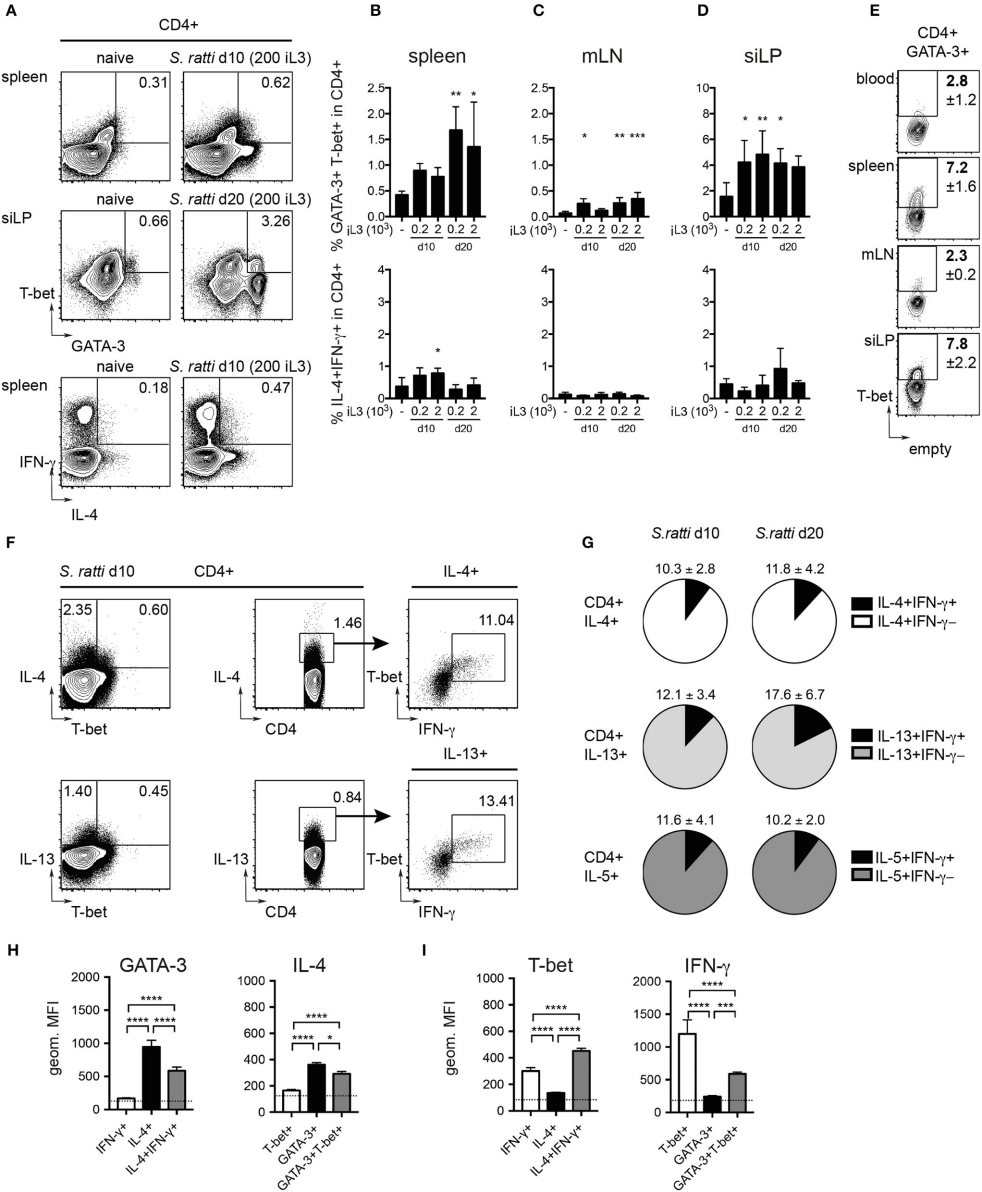
Th2/1 cells with combined Th2 and Th1 features are induced during *S. ratti* infection. Mice infected with 200 or 2,000 *S. ratti* stage 3 larvae were screened for Th2/1 cells co-expressing Th2 and Th1 lineage transcription factors and cytokines. **(A)** Exemplary flow cytometry plots of live CD4^+^ T cells derived from spleen (top and bottom) and small intestinal lamina propria (middle) of an uninfected control and *S. ratti* infected mouse at the depicted days post infection. Numbers indicate frequencies of GATA-3^+^T-bet^+^ and IL-4^+^IFN-γ^+^ cells, respectively. **(B–D)** Frequencies of GATA-3^+^T-bet^+^ cells (top) and IL-4^+^IFN-γ^+^ cells (bottom) within live CD4^+^ T cells isolated from spleen **(B)**, mLN **(C)** and siLP **(D)** as detected after 4h of PMA/ionomycin stimulation. Significance was tested comparing infected and naïve controls. **(E)** Exemplary plots depicting frequencies of T-bet expressing cells in CD4^+^GATA-3^+^ T cells derived from peripheral blood, spleen, mLN and siLP of mice infected for 10 days (200 iL3) stained directly *ex vivo*. Numbers indicate mean values and SD of 4-5 mice. **(F)** Concatenated plots depicting IL-4 single and IL-4/T-bet co-expressing cells (top left) and IL-13 single and IL-13/T-bet co-expressing cells (bottom left) in CD4 cells isolated from spleens of 5 mice infected for 10 days with *S. ratti* (200 iL3). Center and right plots show detection of T-bet^+^ IFN-γ producing cells in the IL-4 and IL-13 producing populations. **(G)** Pie charts depict proportions of IFN-γ^+^ Th2/1 cells (black) within the IL-4 (white), IL-13 (light gray) and IL-5 (dark gray) producing CD4^+^GATA-3^+^ T cell derived from spleens of mice infected with *S. ratti* (200 iL3) for 10 (left) and 20 days (right). Mean and SD of *n* = 5 mice is shown. **(H,I)** Geometric mean fluorescence intensity (MFI) of GATA-3 and IL-4 **(H)** and T-bet and IFN-γ signals **(I)** of the depicted CD4^+^ subpopulations derived from mice infected with *S. ratti* (200 iL3) for 10 days. The MFI of IL-4^−^IFN-γ^−^ and GATA-3^−^T-bet^−^ cells is depicted by a dotted line. Mean and SD of *n* = 5 mice. Data originate from one out of two experiments with similar results. ^*^p < 0.05, ^**^p < 0.01, ^***^p < 0.005, ^****^p < 0.001.

Asking whether infections with *S. ratti* led to the expansion of Th2/1 cells as previously shown for other helminth infections (Peine et al., 2013), we screened the organs of naïve and infected mice for CD4^+^ T cells simultaneously expressing GATA-3 and T-bet. We detected significantly increased frequencies of GATA-3^+^T-bet^+^ Th2/1 cells in all organs of infected mice (Figures 3A–D and SI Figures 2A,B). The proportions of T-bet co-expressing Th2/1 cells within the total GATA-3^+^CD4^+^ population were highest in spleen and siLP of *S. ratti* infected mice (Figure 3E). T-bet/IFN-γ co-expressing cells were detected in similar proportions within IL-4 and IL-13 producers from spleens of mice infected for 10 days (Figure 3F) and the frequencies of IFN-γ^+^ Th2/1 hybrids in Th2 cytokine producing populations were similar when comparing day 10 and 20 post infection (Figure 3G).

As shown previously (Peine et al., 2013), Th2/1 hybrid cells expressed lower levels of GATA-3 and IL-4 than conventional Th2 cells (Figure 3H) and IFN-γ production was significantly decreased compared to Th1 cells (Figure 3I). Notably, T-bet expression by Th2/1 cells was significantly elevated compared to infection-derived Th1 cells (Figure 3I), which might reflect the acute activation status of the Th2/1 cells.

Thereby, our previous demonstration of the induction of Th2/1 hybrid cells in nematode and Schistosome infections (Peine et al., 2013) can be generalized to mice infected with different doses of threadworms.

### Human study population characteristics

To evaluate whether CD4^+^ T cells with combined characteristics of Th2 and Th1 cells also occur in human helminth-infected patients we investigated blood samples of patients infected with *S. stercoralis* and endemic uninfected control donors. The demographic profile of the cohorts is given in Table 1. No differences in age range, gender and socio-economic status were observed between the two studied groups. All individuals were healthy and free of symptoms. Hematological features of both cohorts are depicted in Table 2. No differences concerning hemoglobin levels, red and white blood cell counts, hematocrit or platelet counts were observed between the groups. The frequencies of lymphocytes and eosinophils were significantly elevated in the infected cohort (SI Figures 3A,D), while neutrophil frequencies were significantly lower in *S. stercoralis* infected patients (SI Figure 3B). Blood monocyte and basophil frequencies were not different between the groups (SI Figures 3C,E).

### Human *S. stercoralis* infection is associated with increased Th2 responses

**Figure 4:**
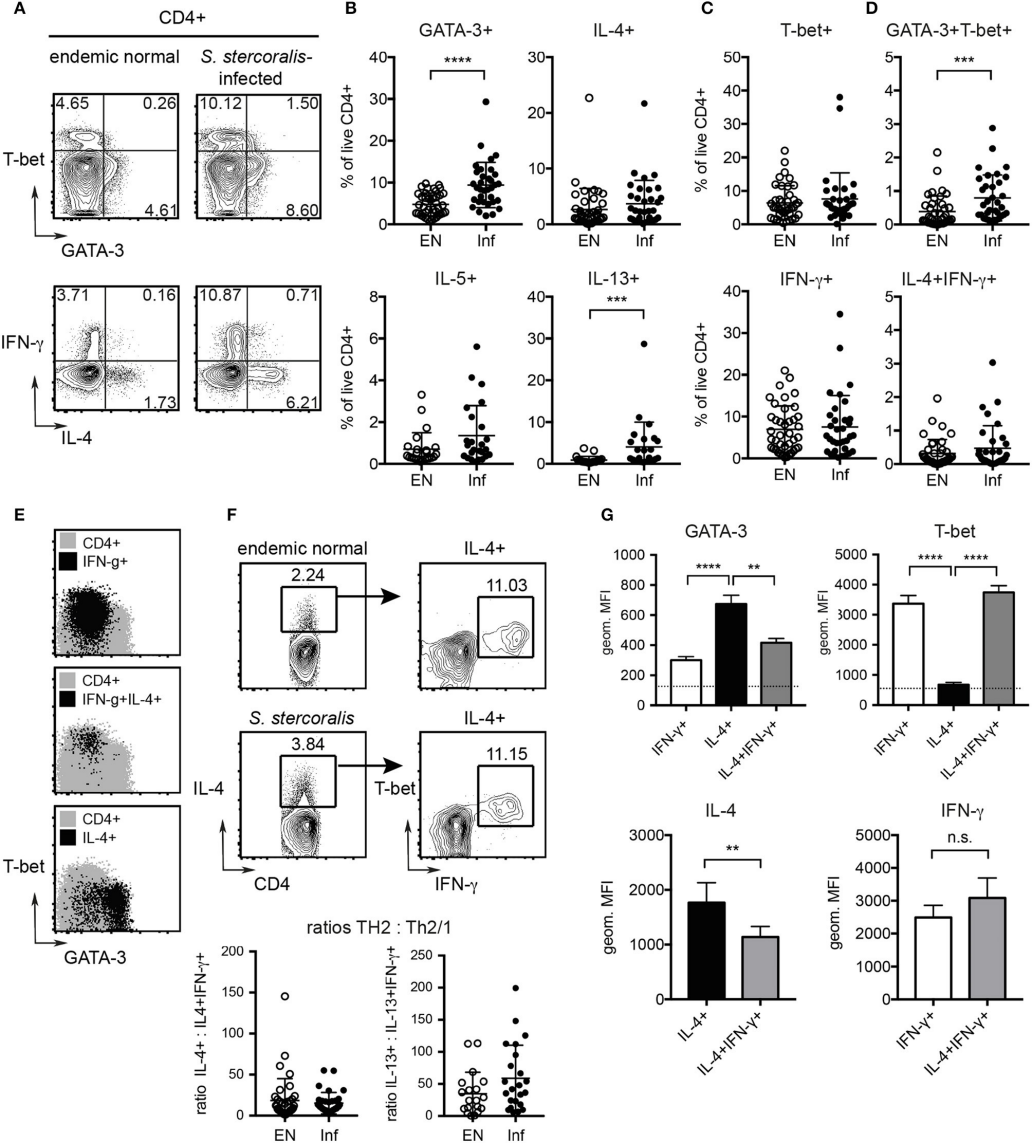
Detection of increased Th2 responses in *S. stercoralis*-infected patients and characterization of human Th2/1 hybrid cells. PBMC where stimulated with PMA/ionomycin and cytokine responses and expression of GATA-3 and T-bet were detected by intracellular staining. **(A)** Exemplary plots of GATA-3 and T-bet (top) and IL-4 and IFN-γ expression (bottom) by CD4^+^ T cells from an uninfected endemic control and a *S. stercoralis* infected donor. **(B)** Frequencies of GATA-3, IL-4, IL-5, and IL-13 expressing Th2 cells as detected in the endemic normal control group (EN) and *S. stercoralis*-infected group (Inf). **(C)** Frequencies of T-bet and IFN-γ expressing Th1 cells. **(D)** Frequencies of GATA-3^+^T-bet^+^ and IL-4^+^IFN-γ^+^ Th2/1 hybrid cells. Mean, SD and individual values are shown. **(E)** Backgating of IFN-γ^+^ Th1, IFN-γ^+^IL-4^+^ Th2/1 and IL-4^+^ Th2 cells (black) on GATA-3 and T-bet expression of total CD4^+^ cells (gray). Exemplary plots of a *S. stercoralis*-infected donor are shown. **(F)** Detection of IFN-γ co-producing cells in the total IL-4^+^CD4^+^ population of a control and *S. stercoralis*-infected patient and ratios of IL-4 single producing:IL-4^+^IFN-γ^+^ cells and IL-13 single producing:IL-13^+^IFN-γ^+^ cells within the cohorts. **(G)** Expression levels of GATA-3 and T-bet in IFN-γ^+^ Th1, IL-4^+^ Th2 and IL-4^+^IFN-γ^+^ Th2/1 cells (top). The MFI of IL-4^−^IFN-γ^−^ cells is depicted by a dotted line. Bottom: Expression levels of IL-4 in Th2 vs. Th2/1 cells and IFN-γ in Th1 vs. Th2/1 cells. Data (mean and SD) are derived from samples of 5 *S. stercoralis* infected donors comprising >100 IL-4^+^IFN-γ^+^ cells assessed in two independent experiments. ^**^p < 0.01, ^***^p < 0.005, ^****^p < 0.001.

To compare the immune status of helminth-infected individuals and uninfected control subjects, PBMC from *S. stercoralis-*infected donors and healthy endemic controls were stimulated with PMA/ionomycin and assessed for markers of Th1 and Th2 differentiation by flow cytometry. The *S. stercoralis-*infected group displayed significantly elevated frequencies of CD3^+^CD4^+^GATA-3^+^ Th2 cells compared to the uninfected control group (Figures 4A,B) and a higher ratio of Th2:Th1 cells (SI Figure 4). While frequencies of IL-4 and IL-5 expressing T cells were similar in both cohorts, the *S. stercoralis*-infected group displayed significantly increased levels of IL-13 expressing cells (Figure 4B). Linear regression analysis confirmed a significant effect of the group in terms of IL-13 levels (*p* = 0.0407) and showed that increased GATA-3 expression was associated with increased IL-13 expression (*p* = 0.0279) (SI Figure 5A). Furthermore, levels of IL-4, IL-13 and IFN-γ significantly increased with age (*p* = 0.0030, *p* = 0.0062, and *p* = 0.0407, respectively, data not shown).

No differences were observed for Th1 cells expressing T-bet and IFN-γ between the cohorts (Figure 4C), but increased T-bet expression levels were associated with higher IFN-γ expression (*p* < 0.0001) (SI Figure 5B).

Hence, *S. stercoralis* infection in humans is marked by Th2 differentiation, while frequencies of Th1 cells are indistinguishable from healthy controls.

### Th2/1 hybrid cells are detectable in blood of *S. stercoralis* infected patients

We next asked whether hybrid Th2/1 cells sharing characteristics of Th2 and Th1 cells were detectable in blood samples of the study cohorts. We detected CD3^+^CD4^+^ T cells distinctly co-expressing IL-4 and IFN-γ in the infected as well as the healthy control group (Figures 4A,D). Furthermore, the *S. stercoralis*-infected cohort displayed significantly increased frequencies of CD3^+^CD4^+^ T cells co-expressing GATA-3 and T-bet compared to healthy controls (Figure 4D). Expectedly, IL-4 single producing cells clustered in the GATA-3^+^ population, while some, but not all IFN-γ^+^ cells expressed elevated levels of T-bet (Figure 4E). IL-4^+^IFN-γ^+^ Th2/1 hybrid cells, however, featured a GATA-3^low^T-bet^high^ phenotype and clustered with the fraction of IFN-γ^+^ cells expressing the highest levels of T-bet (Figure 4E). Investigating the proportions of IFN-γ^+^ cells within the total IL-4^+^ population confirmed that the proportions of Th2/1 cells were similar in uninfected controls and *S. stercoralis* infected patients, hence the ratios of Th2:Th2/1 cells were similar in both cohorts (Figure 4F). However, higher values of conventional Th2 cells (IL-4+, IL-13+ or IL-5+) correlated with increased Th2/1 hybrid cell frequencies co-expressing IFN-γ and the respective Th2 cytokines (*p* < 0.0001) irrespective of the *Strongyloides* infection status (SI Figure 6).

Assessing the expression levels of GATA-3, T-bet, IL-4 and IFN-γ by mean fluorescence intensities (MFI) confirmed that human IL-4^+^IFN-γ^+^ Th2/1 hybrid cells expressed significantly lower levels of GATA-3 and IL-4 than IL-4 (or IL-13, not shown) single producers (Figure 4G). The mean expression levels of T-bet and IFN-γ, however, were similar for IFN-γ single and IL-4^+^IFN-γ^+^ double producing cells (Figure 4G).

Taken together, CD4^+^ T cells with a Th2/1 hybrid phenotype marked by the co-expression of IL-4 and IFN-γ were detectable in *Strongyloides*-infected patients and healthy controls. The frequencies of Th2/1 cells were positively influenced by the magnitude of the overall Th2 response. In contrast to murine Th2/1 hybrids expressing intermediate levels of GATA-3, IL-4 and IFN-γ (Figure 3), human Th2/1 hybrid cells were marked by high IFN-γ production and low GATA-3 expression.

### *S. stercoralis* infection is associated with a distinct serum antibody profile

**Figure 5:**
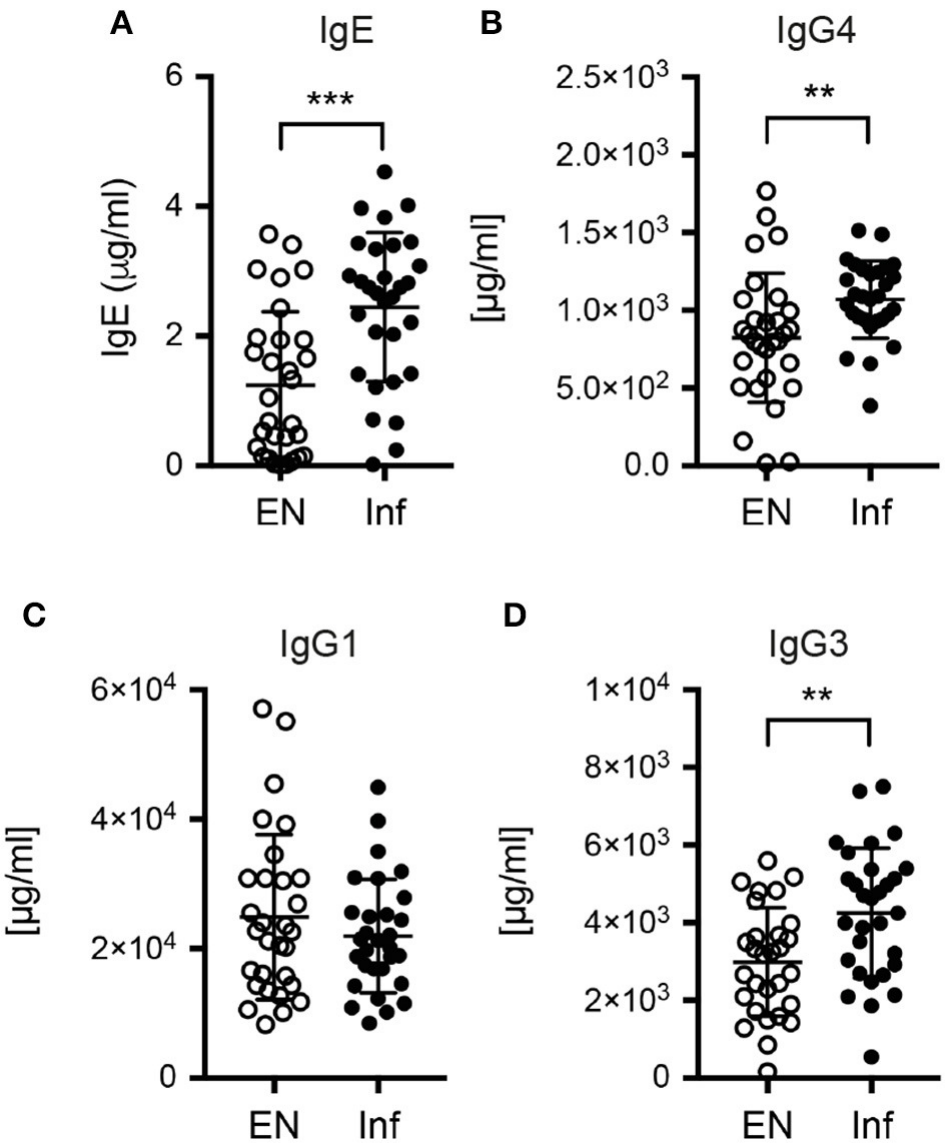
Antibody levels in plasma samples of endemic controls and *S. stercoralis* infected patients. Serum levels of **(A)** total IgE, **(B)** total IgG4, **(C)** total IgG1 and **(D)** total IgG3. ^**^p < 0.01, ^***^p < 0.005.

Next, we assessed if *S. stercoralis* infection was marked by changes in the serum antibody profile. The infected cohort displayed significantly increased total levels of the Th2-associated antibody classes IgE and IgG4 compared to the uninfected control group (Figures 5A,B). IgE levels were positively correlated with IL-13 levels (*p* = 0.0011, data not shown). Interestingly, also the level of IgG3 was significantly increased in the *S. stercoralis*-infected group, while the levels of IgG1, a subclass associated with Th1 responses in humans, were similar in both cohorts (Figures 5C,D).

### Th2/1 cells share effector functions with Th1 cells

**Figure 6:**
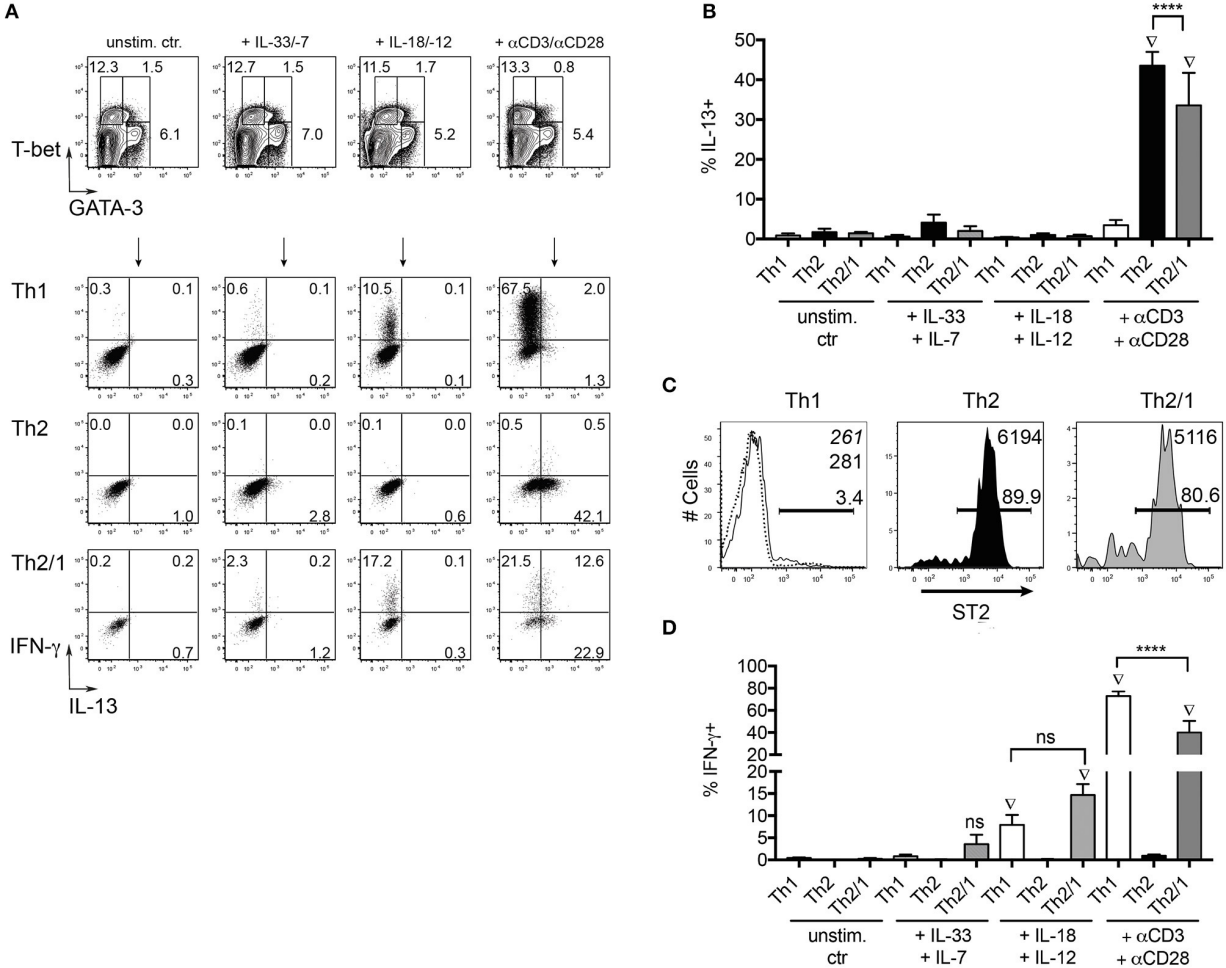
Th2/1 cells share innate function with Th1 cells. **(A)** CD4^+^ cells isolated from *S. ratti*-infected C57BL/6 mice at day 10 post infection with 200 iL3 were stained for GATA-3 and T-bet (top row) after stimulation with IL-33 and IL-7, IL18 and IL-12 or anti-CD3/anti-CD28 antibodies. T-bet^+^Th1, GATA-3^+^Th2 and GATA-3^+^T-bet^+^ Th2/1 cells were assessed for IFN-γ and IL-13 production in response to stimulation (2nd–4th row). Plots are concatenated from five mice. **(B)** Frequencies of IL-13 producers in response to cytokine and TCR-triggering. Mean and SD of 5 *S. ratti*-infected mice is shown. **(C)** ST2 expression by Th1, Th2 and Th2/1 cells from *S. ratti-*infected mice. Mean fluorescence intensities of the populations are given in the right top corner. Dashed line and italic number relate to CD4^+^T-bet^−^GATA-3^−^ cells. **(D)** Frequencies of IFN-γ producers in response to cytokine and TCR-triggering. Data are representative for two independent experiments. ^****^p < 0.001. Diamond symbols indicate values significantly different from the respective unstimulated control samples.

T helper cells not only produce effector cytokines in response to T cell receptor (TCR) triggering by their cognate antigen, but may also react to combinations of IL-1 cytokine family members and STAT activators (Guo et al., 2012). Th2 cells have been shown to produce IL-5 and IL-13 in response to the combined stimulation by IL-33 and IL-7 (Guo et al., 2015), while stimulation by IL-18 and IL-12 results in IFN-γ production by Th1 cells (Robinson et al., 1997; Yoshimoto et al., 1998). We thus asked if Th2/1 hybrid cells induced in helminth infections share innate effector functions with Th2 and Th1 cells. To this end, we isolated CD4^+^ T cells from mice acutely infected with *S. ratti* to compare their TCR-independent and TCR-induced effector responses. In response to IL-33 and IL-7, both Th2 cells and Th2/1 cells failed to produce significant amounts of IL-13 (Figures 6A,B) despite the expression of the IL-33 receptor ST2 (Figure 6C). After stimulation with IL-18 and IL-12, we detected similar IFN-γ responses of by Th1 and Th2/1 hybrid cells (Figures 6A,D).

**Figure 7:**
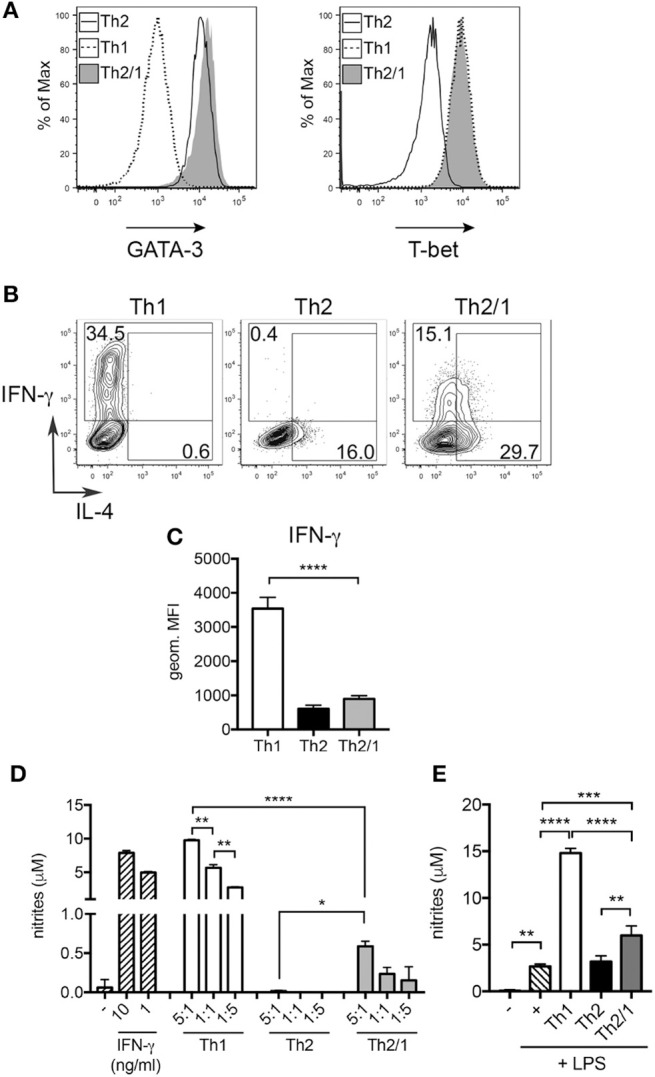
Th2/1 cells are capable of supporting classical macrophage effector function. Murine Th1, Th2, and Th2/1 hybrid cells were generated from naïve CD4^+^ T cells *in vitro* and surveyed for **(A)** GATA-3 (left) and T-bet (right) expression. **(B)** IL-4 and IFN-γ expression by *in vitro*-generated Th1, Th2, and Th2/1 cells in response to PMA/ionomycin stimulation. **(C)** Geometric mean fluorescence intensity of IFN-γ in Th1, Th2, and Th2/1 hybrid cells. **(D)** Nitrite accumulation in supernatant of peritoneal macrophages left untreated (–), stimulated with recombinant IFN-γ or incubated with Th1, Th2, and Th2/1 cells in the depicted T cell:macrophage ratios for 24 h. **(E)** Nitrite concentrations in supernatant of macrophages left untreated (–), stimulated with LPS or co-cultured with T cells (1:1 ratio) in presence of LPS. Mean and SD of five cultures is shown. Data are representative for three independent experiments. ^*^p < 0.05, ^**^p < 0.01, ^***^p < 0.005, ^****^p < 0.001.

We next compared *in vitro* generated murine Th1 and Th2/1 cells for their efficiency in macrophage activation. As shown previously (Peine et al., 2013), naïve cells stimulated in presence of IL-4, IFN-γ and IL-12 differentiated into a homogenous population of Th2/1 hybrids co-expressing GATA-3 and T-bet, many of which co-produced IL-4 and IFN-γ in response to re-stimulation (Figures 7A,B). Similar to Th2/1 cells induced *in vivo* (Figure 3), *in vitro*-generated Th2/1 hybrid cells produced significantly lower levels of IFN-γ than Th1 cells (Figure 7C), despite similar expression levels of T-bet (Figure 7A). In co-cultures with peritoneal macrophages, spontaneous IFN-γ secretion by Th1 cells triggered similar levels of nitric oxide (NO) production by macrophages as induced by IFN-γ added exogenously (Figure 7D). Th2/1 hybrid cells induced approximately 10-fold lower NO-responses (Figure 7D), consistent with their lower IFN-γ production upon stimulation (Figure 7C), while classic Th2 cells failed to trigger an NO response (Figure 7D). However, when macrophages where additionally stimulated with lipopolysaccharide (LPS), the limited amount of IFN-γ produced by Th2/1 cells was sufficient to significantly enhance NO-production over the levels produced in response to the LPS-trigger alone (Figure 7E).

In conclusion, Th2/1 hybrid cells induced in helminth infection are reactive to cytokine triggers leading to TCR-independent IFN-γ production. IFN-γ produced by Th2/1 hybrid cells is sufficient for the classical activation of macrophages under appropriate conditions.

## Discussion

Here we confirm that experimental murine and natural human infections by threadworms are predominantly associated with Th2 responses (Chiuso-Minicucci et al., 2010; Anuradha et al., 2015; Breloer and Abraham, 2016). Mice infected with *S. ratti* displayed locally and systemically increased frequencies of Th2 cells and only low signs of intestinal immunopathology, which is in line with the mostly asymptomatic course of threadworm infections in human patients (Siddiqui and Berk, 2001; Vadlamudi et al., 2006; Montes et al., 2010). *S. stercoralis* infected patients displayed elevated frequencies of blood eosinophils, Th2 cells, IgE and IgG4, while frequencies of Th1 cells expressing T-bet and IFN-γ were similar to uninfected controls.

Previously we have shown that infections with two helminth species, *H. polygyrus* and *S. mansoni*, lead to the differentiation of Th2/1 hybrid cells stably co-expressing GATA-3 and T-bet as well as Th2 and Th1 effector cytokines. This phenotype was maintained after clearance of infection and progression to memory cells, arguing against a transient Th2/1 state (Peine et al., 2013). Here we show that Th2/1 cells also occur in murine experimental threadworm infections, suggesting that Th2/1 hybrid cells are an integral part of the immune response to a wide range of helminth infections.

Th2 and Th1 differentiation have long been considered as mutually exclusive, as cells differentiated under polarizing conditions express distinct patterns of the lineage defining transcription factors GATA-3 and T-bet and the respective cytokines (Zheng and Flavell, 1997; Szabo et al., 2000; Löhning et al., 2002). Furthermore, positive feedback mechanisms and reciprocal inhibition of the developmental programs reinforce and assure the efficient and mutually exclusive differentiation under the appropriate conditions (Ouyang et al., 2000; Afkarian et al., 2002; Jenner et al., 2009). However, not only mice infected with helminths leading to strong Th2 reactions, but also human patients infected with *S. stercoralis* displayed Th2/1 cells co-expressing Th2 and Th1 cytokines, albeit at low levels. To date, we cannot exclude that reprogramming of Th1 cells and/or Th2 cells by opposing signals (Hegazy et al., 2010; Panzer et al., 2012) contributes to Th2/1 generation during helminth infections.

Our murine experimental data show that the proportion of Th2/1 cells within the total GATA-3 expressing Th2 population differed depending on the body compartment. The highest proportions of Th2/1 cells were detected in spleen and small intestinal tissue, while their proportions were lower in gut-draining lymph nodes and blood. We hence speculate that the low frequencies of Th2/1 cells detected in blood of threadworm-infected patients may not necessarily reflect the proportion of Th2/1 cells in other compartments of the human body. Whether Th2/1 hybrids and conventional Th2 cells differ in their proliferative behavior, survival, and reaction to chemokines remains to be established. Further studies are also needed to assess if the preferential location of helminth-induced Th2/1 cells in spleen and the parasite-afflicted organ is associated with specific effector functions.

Our data show that human and murine Th2/1 cells clearly differed phenotypically: while murine Th2/1 hybrids were readily detectable by the coexpression of GATA-3 and T-bet, human Th2/1 cells expressed rather low levels of GATA-3, but were detectable by coexpression of IL-4 and IFN-γ. As not all GATA-3^+^T-bet^+^ cells from helminth-infected mice coproduced Th2 cytokines and IFN-γ simultaneously when restimulated *in vitro* it is important to note that the detection of human hybrids based on the coexpression of cytokines may lead to an underestimation of the proportion of Th2/1 cells in humans. Human and murine Th2/1 cells also differed functionally with respect to IFN-γ production: while human Th2/1 hybrids were marked by high expression levels of IFN-γ, murine Th2/1 hybrids expressed significantly lower levels than Th1 cells. T-bet expression was similar in human Th2/1 cells and Th1 cells, and T-bet expression of murine Th2/1 hybrids even exceeded the levels detected in Th1 cells, which may be explained by differential activation states of the cells. Still, only murine Th2/1 cells were restricted in IFN-γ production, which at least in murine Th1 cells is largely driven and quantitatively controlled by T-bet expression amounts (Szabo et al., 2000; Helmstetter et al., 2015). This functional difference in human vs. murine Th2/1 hybrids might be explained by the finding that human Th2/1 hybrid cells (characterized as IL-4/IFN-γ co-producing cells) displayed rather low levels of GATA-3 expression. It hence seems likely that the lack of GATA-3-driven counter-regulation of T-bet functions allows unabated IFN-γ production by human Th2/1 cells (Chang and Aune, 2007; Jenner et al., 2009). Yet we can only speculate on how human Th2/1 cells retain their (reduced) production of IL-4 and other Th2 cytokines in face of low GATA-3 expression. In mice, conditional deletion of GATA-3 from established Th2 cells diminishes Th2 cell maintenance and IL-5 and IL-13, but not IL-4 production (Pai et al., 2004; Zhu et al., 2004). Whether other factors such as c-Maf expression allow human hybrids to express Th2 cytokines in a setting of low GATA-3 expression remains to be investigated (Kim et al., 1999). Similarly, it remains to be established if murine and human CD4^+^ T cell co-expressing Th2 and Th1 cytokines differ in other regulatory elements of effector functions and if their relatively high proportion in the spleen has functional relevance.

An interesting finding in our patient survey was that not only Th2-associated antibody classes (IgE, IgG4) were elevated in threadworm-infected patients, but also IgG3 was significantly increased in the infected cohort. IgG3 is a potent pro-inflammatory antibody with high affinity to the activating Fcγ receptor I (FcγRI), the latter being central for the control of bacterial infections (Ioan-Facsinay et al., 2002; Vidarsson et al., 2014). Both IgG3 class switching by B cells and FcRI expression by human myeloid cells are positively regulated by IFN-γ (Erbe et al., 1990; Snapper et al., 1992). Hence IFN-γ expression by Th2/1 cells in reactive lymph nodes or the spleen, where their proportion is relatively high, might contribute to the diversification of antibody responses and assure that antibodies primarily involved in opsonization of pathogens or their secreted components do not go short in Th2-associated infections mainly driving IgE and IgG4 production by B cells.

We found that murine Th2/1 cells not only co-express IFN-γ and Th2 cytokines in response to TCR-triggering, but also responded to the combination of the STAT activator IL-12 and IL-1 family member IL-18 with production of IFN-γ. It is tempting to speculate that the differentiation of Th2/1 cells in response to helminths inducing strong Th2 reactions, such as *S. ratti, H. polygyrus* and *S. mansoni*, may provide an advantage to the infected host in preventing an overtly and exclusively Th2-biased immune set-up. It is critical for the immune system to quickly respond to rapidly replicating microbes controlled by Th1 responses and classically activated macrophages. Similar to e.g. enhanced IFN-γ production by NK cells sensing IL-18 and IL-12 during infection with the protozoan *Toxoplasma gondii* (Cai et al., 2000), Th2/1 cells co-induced with conventional Th2 cells during helminth infections and co-infiltrating the infected organs may contribute to early IFN-γ production in response to cytokine triggers produced upon protozoan, bacterial and viral infections. Although most likely subordinate to Th1 cells in this regard, their activated state during ongoing helminth infection may facilitate IFN-γ responses by Th2/1 cells in response to cytokine triggers and hence help in supporting adequate Th1 reactions in face of ongoing Th2 responses.

There is experimental evidence that nitric oxide is toxic for *Strongyloides* parasites and limits worm fecundity as well as autoinfection in mice (Ruano et al., 2012, 2015). We show that murine Th2/1 cells are, expectedly, subordinate to Th1 cells in driving NO production by macrophages, coinciding with their limited IFN-γ production. However, in conjunction with TLR triggering, the limited IFN-γ production of Th2/1 cells was sufficient to stimulate significant NO production. Hence Th2/1 cells may be important to ensure that the helminth-infected host is able to quickly and adequately react to co-infections where an unabated IFN-γ response is crucial. Furthermore, IFN-γ production by Th2/1 cells generated during helminth infection often including tissue migratory larval stages may be advantageous in preventing bacterial dissemination facilitated by tissue damage (Pesce et al., 2008), especially upon parasite re-exposure of organs densely seeded with Th2 memory cells (Steinfelder et al., 2017). We currently assess if Th2/1 cells constitute a considerable source of IFN-γ and of other factors affecting the immune response to the parasite and, possibly, coinfecting pathogens.

Taken together we provide evidence that Th2/1 cells developing during helminth infections are not a feature restricted to the murine host, but also occur in humans. Especially human Th2/1 cells seem to be able to produce IFN-γ in similar amounts as Th1 cells, posing the question whether they may efficiently counter-regulate an overt Th2 bias in helminth infections and allergic diseases. By sharing effector functions with Th1 cells they may act as a provision against hampered responses to co-infecting pathogens in face of a strongly Th2-biased immune status.

## Author contributions

CB and SR performed all the experiments. SH and SR conceptualized and designed the research. SB, AR and YB recruited and classified human subjects. CB, MLB, AK, and RM analyzed the data. CB, MB, SH, and SR wrote the manuscript. All authors approved the final version of the manuscript.

### Conflict of interest statement

The authors declare that the research was conducted in the absence of any commercial or financial relationships that could be construed as a potential conflict of interest.
