# Supplementary material for: Th2/1 Hybrid Cells Occurring in Murine and Human Strongyloidiasis Share Effector Functions of Th1 Cells
Source: Front Cell Infect Microbiol. 2017 Jun 20;7:261. doi: 10.3389/fcimb.2017.00261 (PMC5476698; doi:10.3389/fcimb.2017.00261)
Supplement: Supplementary file 1 [file Presentation1.pdf]

Supplementary Material

Th2/1 hybrid cells occurring in murine and human strongyloidiasis share effector functions of Th1 cells

Cristin N. Bock[1], Subash Babu[2,3], Minka Breloer[4], Anuradha Rajamanickam[2], Yukhti Boothra[2], Marie-Luise Brunn[4], Anja A. Kühl[5], Roswitha Merle[6], Max Löhning[7,8], Susanne Hartmann[1], Sebastian Rausch[1*]

*** Correspondence:** Sebastian Rausch, sebastian.rausch@fu-berlin.de

SI Fig. 1 (related to Fig. 2)

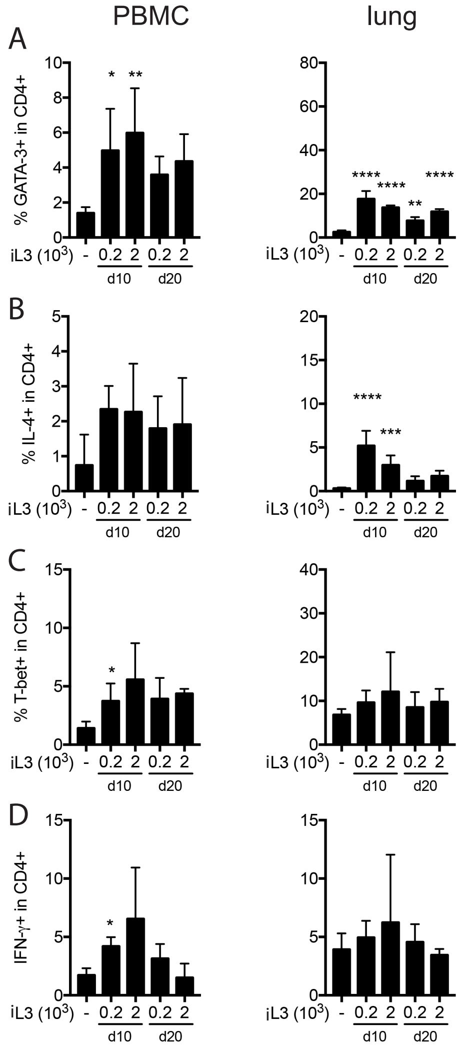

SI Fig. 1. ***S. ratti*** **infection leads to systemic and local Th2 response.**

Phenotypes of $CD4^+$ T cells were assessed in mice infected with 200 or 2000 *S. ratti* iL3. Frequencies of **(A)** GATA-3^+, **(B)** IL-4^+, **(C)** T-bet^+ and **(D)** IFN-γ^+ cells within live $CD4^+$ T cells isolated from peripheral blood (left) and lung tissue (right). Mean + SD of n=5-6 (naïve ctr.) and 4-5 (infected) mice. Data from one out of two experiments with similar results are shown. * $p < 0.05$, ** $p < 0.01$, *** $p < 0.005$, **** $p < 0.001$ comparing infected to naïve controls.

SI Fig. 2 (related to Fig. 3)

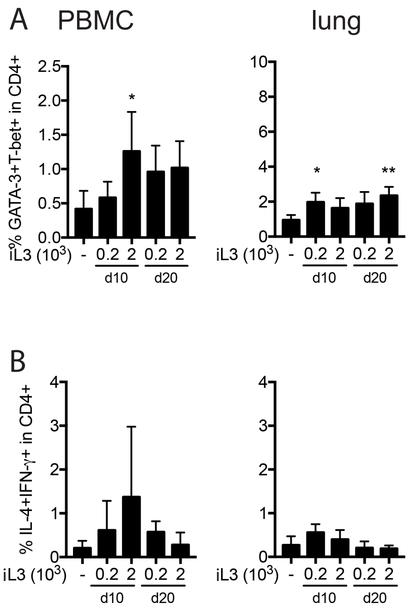

SI Fig. 2. Th2/1 cells with combined Th2 and Th1 features are induced during *S. ratti* infection.

Mice infected with two doses of *S. ratti* were screened for Th2/1 cells co-expressing Th2 and Th1 lineage specifying transcription factors and cytokines. Frequencies of **(A)** GATA-3^{+}T-bet^{+} cells and **(B)** IL-4^{+}IFN-γ^{+} cells within live CD4^{+} T cells isolated from peripheral blood (left) and lung tissue (right) as detected after 4h of PMA/ionomycin stimulation. Significance was tested comparing infected and naïve controls. Mean and SD of n=4-6 mice. Data originate from one out of two experiments with similar results. * $p < 0.05$, ** $p < 0.01$.

SI Fig. 3

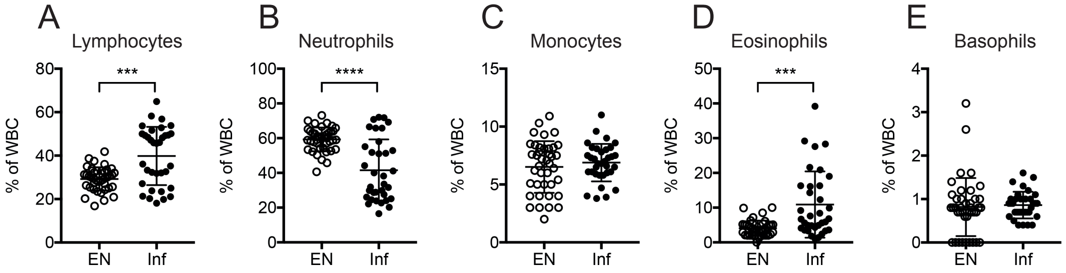

SI Fig. 3. *S. stercoralis*-infected patients have increased lymphocyte and eosinophil frequencies in peripheral blood while neutrophils are decreased. Frequencies of **(A)** lymphocytes, **(B)** neutrophils, **(C)** monocytes, **(D)** eosinophils and **(E)** basophils in white blood cells of endemic uninfected controls (EN) and *S. stercoralis*-infected patients (Inf). *** $p < 0.005$, **** $p < 0.001$.

SI Fig. 4 (related to Fig. 4)

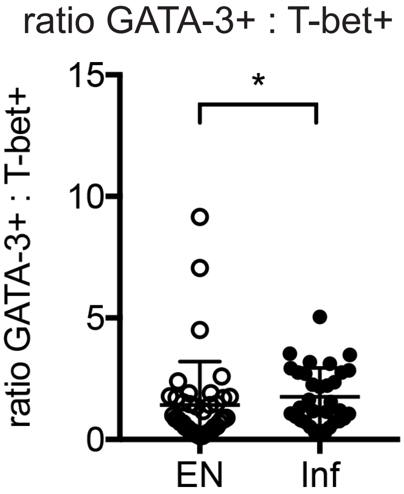

SI Fig. 4. Th2:Th1 ratio is increased in *S. stercoralis*-infected patients.

$CD3^+CD4^+$ cells were assessed for frequencies of GATA-3- and T-bet-expressing cells and ratios of Th2 cells : Th1 cells were calculated. * $p < 0.05$.

SI Fig. 5

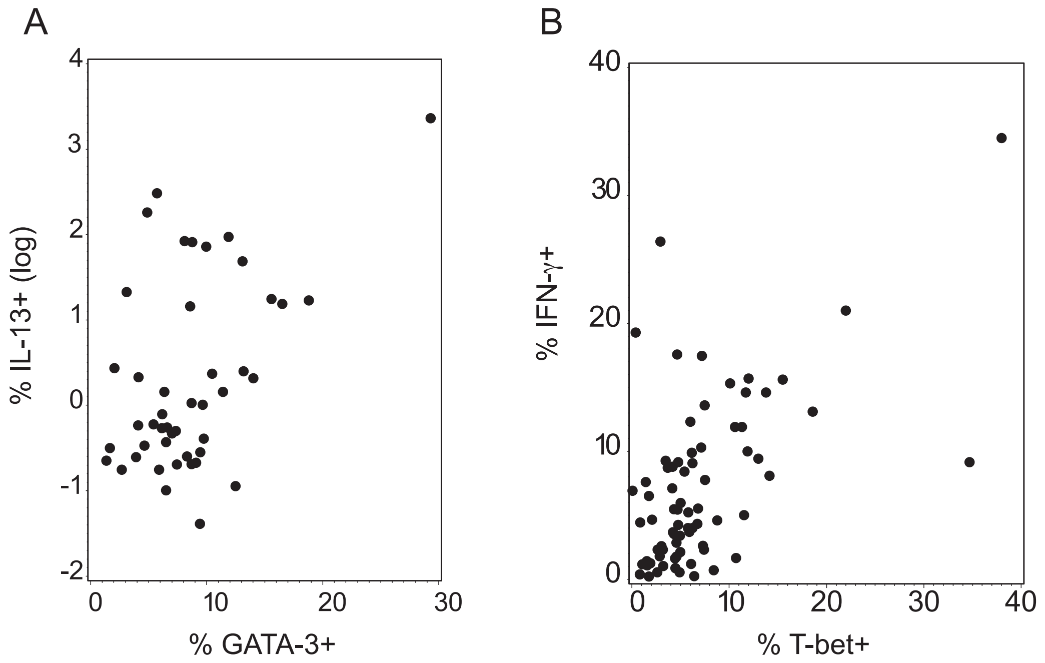

SI Fig. 5. Correlations between lineage transcription factor and cytokine expression of human $CD4^+$ T cells. Significant positive correlation between **(A)** GATA-3 and IL-13 expression ($p=0.0279$) and **(B)** T-bet and IFN-γ ($p<0.0001$) expression.

SI Fig. 6

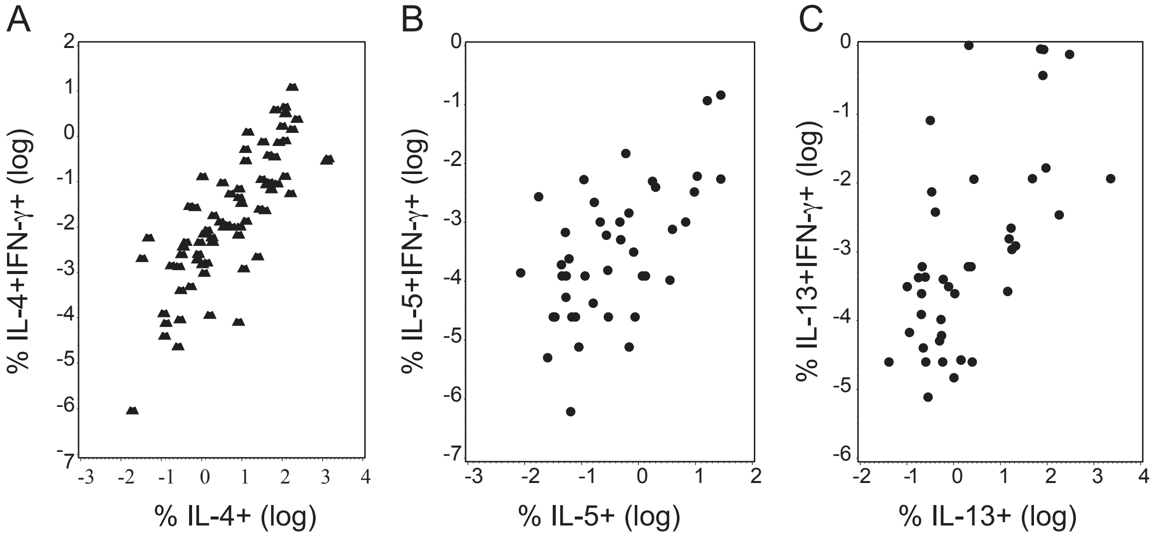

SI Fig. 6. Correlations between frequencies of human Th2 and Th2/1 hybrid cells. Significant positive correlation between frequencies of **(A)** IL-4+ and IL-4+IFN-γ^+ cells, **(B)** IL-5+ and IL-5+IFN-γ^+ cells and **(C)** IL-13+ and IL-13+IFN-γ^+ cells. $p<0.0001$.

frontiers
